# Review of "Mining Imperfect Data" by Ronald K. Pearson

**DOI:** 10.1186/1475-925X-4-43

**Published:** 2005-07-18

**Authors:** Francisco Azuaje

**Affiliations:** 1 Computer Science Research Institute, University of Ulster, Jordanstown, Co. Antrim, BT37 0QB, Northern Ireland, UK

## 

*Imperfect *data are prevalent in health informatics and biomedical engineering. Therefore, data analysis and modelling tools in real world applications must be able to represent, recognise and process imperfect data. Several studies have aimed to classify the different types of data imperfections and their possible sources. Incompleteness, imprecision, inconsistency and uncertainty are some of the problems associated with data imperfection [1]. In data-driven application domains, such as bioinformatics and medical informatics, these types of problems may originate from unreliable data acquisition sources, faulty sensors, data collection errors and the lack of data representation standards. Although these factors and constraints are widely accepted by the health informatics and data mining communities, most applications have traditionally ignore the need for developing appropriate approaches to representing and reasoning with imperfect data.

Ronald Pearson's book discusses the detection of different types of imperfect data, their potential sources, implications and methods to treat them. It mainly focuses on three types of data imperfections: Outliers, missing data and misalignments. The introductory chapter defines these anomalies, presents a rationale for their analysis and overviews important approaches to pre-processing and detection. The second chapter is a deeper discussion of these types of imperfections, their causes and consequences for data analysis. The third chapter describes techniques for detecting univariate outliers followed by a chapter on data pre-processing, which covers techniques for dealing with missing data, time-series and multivariate outliers. Chapter 5 concentrates on the application of functional equations and inequalities to characterise data. Chapter 6 further discusses the generalised sensitivity analysis (GSA) framework, which is one of the key data analysis tools applied in the book. Pearson summarises the basis for GSA with the following statement: "A 'good' data analysis result should be insensitive to small changes in either the methods or the datasets on which the analysis is based". Chapter 7 describes different data sampling strategies that may be applied to implement GSA. The last chapter discusses some of the challenges and open questions for mining imperfect data. This book emphasises the application of boxplots for summarising, visualising and comparing results. Examples are illustrated using real data sets relevant to medicine, bioinformatics and industrial applications.

The book provides the reader with clear descriptions and accessible discussions of problems, motivations, methods and interpretations. The author excels when describing the importance of tools and their applications. The first chapter is a prime example of how to introduce important concepts and methods to a wide readership that may be composed by students, researchers and non-specialists.

The book should not be seen as a comprehensive, detailed description of methods for dealing with different types of data imperfections. For example, with regard to missing value estimation, it might be complemented by Little and Rubin's *Statistical Analysis With Missing Data *[2] or Allison's *Missing Data *[3]. *Statistical Analysis and Data Display *by Heiberger and Holland [4] may be a relevant reference for summarising and comparing data analysis results [5]. Although the chapter on functional equations is important, I would have expected a stronger rationale for stressing this particular approach. The final chapter offers an interesting discussion on key challenges for mining imperfect data. Nevertheless, readers could have been benefited from a concluding chapter that also summarises the author's suggestions on when and why specific approaches should be applied. Moreover, the discussion on analysing large datasets could be enriched by including other, more recent investigations from the areas of data mining and visualisation, as well as different applications domains, such as bioinformatics.

Having said that, it is necessary to highlight that this book represents a significant contribution, which should benefit anyone interested in developing or assessing data mining and machine learning applications in medicine and biology. I will certainly keep it as a key reference and recommended reading for final year undergraduate and postgraduate students.
